# Psychological Flexibility Mediates Wellbeing for People with Adverse Childhood Experiences during COVID-19

**DOI:** 10.3390/jcm11020377

**Published:** 2022-01-13

**Authors:** Angela Browne, Owen Stafford, Anna Berry, Eddie Murphy, Laura K. Taylor, Mark Shevlin, Louise McHugh, Alan Carr, Tom Burke

**Affiliations:** 1 School of Psychology, University College Dublin, D04 V1W8 Dublin, Ireland; angela.browne1@ucdconnect.ie (A.B.); owenstafford@ucd.ie (O.S.); annanibheara@gmail.com (A.B.); eddie.murphy2@hse.ie (E.M.); laura.taylor@ucd.ie (L.K.T.); louise.mchugh@ucd.ie (L.M.); alan.carr@ucd.ie (A.C.); 2 Health Service Executive, CHO 8 (Laois/Offaly), R32 YFW6 Laois, Ireland; 3 School of Psychology, Queen’s University Belfast, Belfast BT7 1NN, UK; 4 School of Psychology, University of Ulster, Belfast BT1 6DN, UK; m.shevlin@ulster.ac.uk; 5 School of Psychology, National University of Ireland, H91 CF50 Galway, Ireland

**Keywords:** COVID-19, pandemic, adverse childhood experiences, ACEs, psychological flexibility, wellbeing, mental health, psychological distress, Ireland

## Abstract

Background: The psychological impact of COVID-19 is multifaceted, both acute and chronic, and has not affected everyone equally. Method: This longitudinal study compared those with and without Adverse Childhood Experiences (ACEs) on measures of psychological distress and wellbeing over time. Results: All groups (No ACE, Low ACE, and High ACE) had similar levels of distress at Time 1, with significant increases in psychological distress for those with ACEs over time, but not for those without. Psychological Flexibility was strongly and significantly associated with decreases in psychological distress and improved wellbeing. It significantly mediated the relationship between ACE and wellbeing. Conclusions: Those with ACEs report significantly increased psychological distress over time, compared to those without ACE during the COVID-19 pandemic. Evidence-based interventions using Psychological Flexibility may improve mental health and wellbeing to help further mediate its effects.

## 1. Introduction

Since the World Health Organisation (WHO) declared a global pandemic on the 11th of March 2020, due to the rapid spread of a novel virulent strain of coronavirus (COVID-19), there have been 228.18 million recorded confirmed cases worldwide and 4.69 million COVID-19 related deaths as of the 18th of September 2021. To contextualize this within the context of other 21st century infectious diseases, the Severe Acute Respiratory System (SARS) pandemic of 2002/2003 infected 8098 people worldwide, of which 774 died, while an estimated 123,000–203,000 people died worldwide due to Swine flu (H1N1 Influenza) in 2009/2010. Only the Spanish flu over a hundred years earlier (1918) is comparable in terms of deaths and numbers infected.

Since the pandemic began, Ireland, like many other countries, has engaged in both national and regional stepped restrictions to limit the spread of the virus [1,2,3]. While these measures were deemed necessary for public health and safety, research from previous pandemics and evolving research from the present pandemic has highlighted the adverse psychological and physical effects such actions can have, in the acute phase and the longer term, for both those infected and those who did not contract the virus [4,5,6,7,8].

The consensus within the literature to date suggests social distancing and other imposed measures of social distancing are strongly associated with depression, anxiety and stress, psychotic symptoms, e.g., paranoia and hallucinations, and reduced subjective wellbeing [9,10,11,12,13,14,15,16,17]. By way of example, Hyland et al. [18] found that more than one in four (27.7%) people surveyed during lockdown measures in Ireland screened positive for Generalized Anxiety Disorder or Depression. For some, the uncertainty and fear perpetuated by living for a prolonged period alongside a deadly virus, in addition to living with increased psychological stressors (i.e., unemployment; lack of childcare, working from home) and reduced availability of routine coping mechanisms (i.e., social support; work) can cause chronic stress and consequently negatively influence mental health and wellbeing [1,5,19]. However, the new-onset acute and chronic stressors associated with COVID-19 do not impact all individuals equally [7,20,21,22,23]. The stress sensitization hypothesis [24] posits that adversities in early childhood sensitize individuals to subsequent proximal stress and increases the risk for psychopathology in the face of future stressful life events. As such, one such population that is likely to be vulnerable to the stressors associated with COVID-19 are individuals who have experienced Adverse Childhood Experiences (ACEs) [25,26,27].

ACEs can be broadly defined as adverse traumatic experiences which occur during the first eighteen years of life, and are usually categorized into physical and sexual abuse/neglect, emotional abuse/neglect, and household dysfunction [28]. While the effect of these adversities on an individual is multifaceted, research suggests a strong graded relationship between the number of ACEs experienced, and lifelong physical and psychological ill-health [28,29,30,31]. Examples of ACEs include sexual, emotional, and physical abuse, neglect, parental mental illness, substance abuse, parental separation, and criminal behaviour; all of which can be reported through the Adverse Childhood Experiences questionnaire [28].

Early research has indicated that those who experienced ACEs are more vulnerable to both the direct and indirect effects of COVID-19, than those without [26,27,32,33,34,35,36,37,38,39]. In addition, those with several ACEs (i.e., ≥4) are more likely than individuals without ACEs to have existing mental health difficulties, chronic physical ill-health, and are disproportionately from lower socioeconomic backgrounds [21,25,28,40,41,42,43]. Based on the stress sensitization hypothesis, COVID-19 is an additional major stressor to individuals with an already heightened liability to physical and psychological difficulties [26,44], with comorbid medical conditions shown to relate to elevated psychological distress, notwithstanding ACEs [14,21,23,45].

There is recent research on mitigating the effects of COVID-19 and improving mental health and wellbeing. Interventions that promote Psychological Flexibility, such as Acceptance and Commitment Therapy (ACT) and Mindfulness are shown to be effective [19,46,47,48], and also mitigate the effects of ACEs [49,50]. Psychological Flexibility, the ability to adapt to situations by accepting and fully experiencing all thoughts and feelings and engaging in value-based behaviour, aims to promote positive mental health and wellbeing and reduces psychological distress [51]. Conversely, a lack of Psychological Flexibility can be present alongside psychological processes such as rumination in depression, avoidance in anxiety, and other alternations in executive functioning in neurodevelopmental disorders such as schizophrenia [14,52,53,54]. What is less known is to what extent does Psychological Flexibility mediate the relationship between ACEs and psychological distress secondary to COVID-19.

Much of the research to date on mental health and wellbeing concerned with COVID-19 have used cross-sectional data [10]. While this is important, the lasting impact and chronicity of stress experienced, especially when considering vulnerable populations, requires longitudinal investigations. By understanding the immediate and longer-term psychological impact of COVID-19, services could better direct and understand responses following the immediacy of the pandemic. Research by Holmes et al. [55] has proposed that investigations into the effects of COVID-19 on these vulnerable groups should be an immediate priority.

This study aims to examine the effect of prolonged stress on an Irish cohort’s mental health and wellbeing during over a 10-month period and is specifically interested in the response profile and reported stress of people who have experienced ACEs. This study hypothesized that the reported psychological stressors resulting from COVID-19 will increase over time for those with ACEs, relative to the control population, based on the stress sensitisation hypothesis [24,44]. Consequently, it was hypothesized that those with no ACEs will have significantly lower stress at Time 2 when compared to those with ACEs. Finally, this study aims to investigate whether self-reported Psychological Flexibility is a protective factor that can help mitigate the negative impacts of ACEs and psychological distress on wellbeing over time, due to the known positive relationship between psychological wellbeing and psychological flexibility [46,47,48,49,50].

## 2. Materials and Methods

### 2.1. Participants

This study employed a longitudinal prospective follow-up survey design. Participants were recruited through national media outlets, social media, and professional networking websites in Ireland, during the first period of public ‘Level 5 Lockdown’ in Ireland (27 March to 8 June 2020), to assess the psychological impact of COVID-19 [9]. Participants were asked to follow a link to access the study information sheet, consent, and questions via Qualtrics. Once consent was obtained, participants completed the outcome measures. This study received approval from the host research ethics committee.

Of the cohort who completed the cross-sectional aspect of this study, *n* = 450 participants consented to be contacted prospectively via email for follow-up and *n* = 231 provided contact details for follow-up. These data were collected during a repeated ‘Level 5 Lockdown’ in Ireland (January 2021), with the same government restrictions in place as when original data was collected. The inclusion criteria for the study were being over the age of 18 and living in Ireland. The final sample consisted of N = 231 (88% female), consistent with the gender distribution of the original cross-sectional study [9,23], with participants aged between 20 and 76 years. Approximately 54% of participants earned within €25,000–74,999, with 85% having completed a university degree. 50% of sample were married, 35% in committed relationships, 9% were engaged, and 6% single. Overall, 41% of the sample had children.

### 2.2. Measures

Six questionnaires were used in this study, with four administered across both time-points, i.e., the Depression, Anxiety and Stress Scale (DASS-21, [56]); Warwick-Edinburgh Mental Wellbeing Scale (WEMWBS, [57]); Brief Illness Perception Questionnaire (BIPQ, [58]); and the Effects of COVID-19 Questionnaire (ECQ, [9]). Two questionnaires were included at the follow-up assessment—The Adverse Childhood Experiences (ACEs) Questionnaire [28] and the PsyFlex [59].

#### 2.2.1. Depression, Anxiety, and Stress

The Depression, Anxiety and Stress Scale-21 (DASS-21) comprises of three subscales, with each subscale measuring self-reported depression, anxiety, and stress. Each subscale contains seven items, with a 4-point Likert scale (0–3) and Depression, Anxiety and Stress scores are calculated by summing with possible scores ranging from 0 to 21. A recent systematic review indicated acceptable psychometric properties of the DASS-21 scores [60]. Cronbach’s alpha values of 0.81 and 0.89 are report for the Depression and Anxiety subscales, respectively. The alpha value for the Stress subscale was observed at 0.78, which is considered “fair” [61].

#### 2.2.2. Mental Wellbeing

The Warwick-Edinburgh Mental Wellbeing Scale (WEMWS) is a 14-item measure covering both hedonic and eudaimonic facets of mental health. The positively worded items capture various concepts of wellbeing, including positive affect, psychological functioning, and interpersonal relationships. The scale uses a 5-point Likert scale (1–5) producing possible scores ranging from 14 to 70, with higher scores indicative of greater wellbeing. Stewart-Brown et al. [62] reported sound psychometric properties for the WEMWS in diverse populations; the reliability of the WEMWBS is noted to be “good” within a student sample, with an observed Cronbach’s alpha of 0.89 [61].

#### 2.2.3. Illness Perception

The Brief Illness Perception Questionnaire is a seven-item scale designed to assess both the emotional and cognitive representation of illness, adapted for use with COVID-19 [9,23]. The items are responded to on a 11-point scale (0–10) producing possible scores ranging from 0 to 70, with higher scores indicative of greater levels of perceived illness. Broadbent, Petrie, Main, and Weinman (2006) reported acceptable levels of reliability and validity [58], with a “good” Cronbach’s alpha value of 0.85 reported [61].

#### 2.2.4. COVID-19 Specific Stress

The Effects of COVID-19 Questionnaire is a 34-item tool designed to measure individuals’ perception of COVID-19 related stresses and associated gratitude. This measure contains four subscales: Personal Stress (items 1–13), Parenting Stress (items 14–21), Older Aging Parent Stress (22–25), and Gratitude (26–34), in which respondents must choose from five response options. Within the ECQ subscales, the ranges for Personal Distress are: Normal 0–12; Mild 13–19; Moderate 20–26; Severe 27–33; Extremely Severe ≥34. Burke et al. [9] reported satisfactory levels of reliability for this scale with a Cronbach’s alpha of 0.79 for the Personal Distress subscale.

#### 2.2.5. Adverse Childhood Experiences

The Adverse Childhood Experiences (ACEs) Questionnaire is a 10-item self-report scale used to measure childhood trauma. Each question asks about experiences growing up during the first eighteen years of life and provides either a “yes” or “no” answer. If a response is yes, a score of 1 is given, with scores ranging from 0–10, which can be used to quantify the number of ACES experienced by that individual. A Cronbach’s alpha of 0.88 has been reported for the ACE Questionnaire [63].

#### 2.2.6. Psychological Flexibility

The PsyFlex questionnaire is a 6-item self-report scale measuring the process of Psychological Flexibility. It assesses psychological flexibility in a state form with high temporal specificity, asking about experiences in the past week. Each item is rated on a 5-point scale from 1 (very often) to 5 (very seldom), producing a possible range of scores from 6 to 30, where higher scores indicate higher psychological flexibility. The PsyFlex has a one-factor structure with excellent reliability (Raykov’s r = 0.91), as well as evidence of convergent, divergent, and incremental validity [51,59]. Cronbach’s alpha for the current study was observed at 0.87 for the total cohort (*n* = 231); 0.85 for the No ACE cohort (*n* = 75); and 0.88 for the cohort with self-reported ACE (*n* = 156).

### 2.3. Statistical Analyses

Participants were stratified as those with No ACE (0), Low ACE (1–3) and High ACE (≥4) based on the ACE Questionnaire. The mean differences between Time 1 and 2 were tested using paired t-tests, and Cohen’s d effect size was also estimated (small = 0.20, medium, 0.50, large 0.80; (Cohen, 1988). Mixed ANOVAs (2 (Time) x 4 (ACE group)) were used for comparisons between groups and within groups on continuous variables (DASS-21, and WEMWBS), with multiple comparisons considered. Pearson product-moment correlations were used to determine associations between variables. Mediation analyses were conducted using Hayes [64] PROCESS macro for SPSS, to look at the effects of childhood stressors (perpetuating factor) and psychological flexibility (protective factor) on the longitudinal psychological outcomes of people during COVID-19 in terms of psychological stress (DASS-21) and wellbeing (WEMWBS).

### 2.4. Role of Funding Sources

This research was in part funded and supported by the Health Research Board and Irish Research Council (COV19-2020-044). The funder had no role in study design, data collection, data analysis, data interpretation, or writing the manuscript.

## 3. Results

### 3.1. Changes within Each Group

Did mean levels of psychological distress change significantly from Time 1 to Time 2, and differently by groups?

Changes on mental health and well-being measures for the total cohort (*n* = 231), and then within each ACE group (No ACE (*n* = 75); Low ACE (*n* = 110); and High ACE (*n* = 46)) from Time 1 to Time 2 were examined. As seen in Table 1, there were no significant changes in mean scores for Depression, Anxiety, or Stress for those with No ACE over time, as measured by the DASS-21. However, those with Low ACE scores had significant increases in all areas of psychological distress from Time 1 to Time 2—Depression (t(109) = 5.60, *p* < 0.001, d = 0.53), Anxiety (t(109) = 2.18, *p* = 0.032, d = 0.21) and Stress (t(109) = 4.60, *p* < 0.001, d = 0.44). Furthermore, individuals with High ACE had a significant increase in Depression scores (t(45) = 3.29, *p* = 0.002, d = 0.49). In terms of relative change, repeated measure ANOVA revealed a significant group-by-time interaction effect (F (2228) = 3.68, *p* = 0.027) for Depression. A one-way ANOVA revealed no significant differences in depression scores between groups at Time 1 (F (2228) = 0.036, *p* = 0.964) but significant differences between groups at Time 2 (F (2228) = 3.11 *p* = 0.046). Planned contrasts (Dunnet T) to test the hypothesis that those with No ACE would score lower than those with ACEs in the analysis, indicated that the increase in Depression scores in the No ACE group (*M* = 5.93, SD = 4.77) was significantly lower (*p* < 0.05) than the corresponding changes within the Low ACE (*M* = 7.75, SD = 5.48) and High ACE groups (*M* = 7.87, SD = 5.68).

When Anxiety was considered within the repeated measure ANOVA, there was no significant group-by-time interaction effect (F (2228) = 2.35, *p* = 0.098) for anxiety. One-way ANOVAs revealed no significant differences in scores at Time 1 or Time 2 between groups (*p* > 0.05).

A final repeated measure ANOVA revealed a significant group-by-time interaction effect (F (2228) = 4.27, *p* = 0.015) for Stress. A one-way ANOVA revealed no significant differences in stress scores between groups at Time 1 (F (2228) = 0.025, *p* = 0.975) but revealed significant differences at Time 2 (F (2228) = 3.21, *p* = 0.042, partial *n*^2^ = 0.027). Planned contrasts indicated that the increase in stress scores for those with No ACE (*M* = 6.69, SD = 5.01) was significantly lower (*p* = 0.011) than those with Low ACE (*M* = 8.61, SD = 8.61) but not significantly lower (*p*= 0.168) than those with High ACE scores (*M* = 7.87, SD = 4.60).

Interestingly, there was no significant change within any group on mean levels of COVID-19 specific illness perception over time, as measured by the BIPQ.

Did mean levels of wellbeing change significantly from Time 1 to Time 2 and differently for groups?

Mean levels of wellbeing decreased significantly for all groups over time—the whole sample (*p* < 0.001, d = 0.367); those with No ACE (*p* < 0.001, d = 0.266); those with Low ACE (*p* < 0.001. d = 0.404); and those with High ACE (*p* < 0.001, d = 0.452). In contrast, there were no significant changes, positively or negatively, within any group when comparing gratitude over time.

### 3.2. Mediational Analysis

Does ACE exposure have a perpetuating effect on psychological symptoms during periods of prolonged stress, and is psychological flexibility protective and a mediator of this relationship?

**Figure 1:**
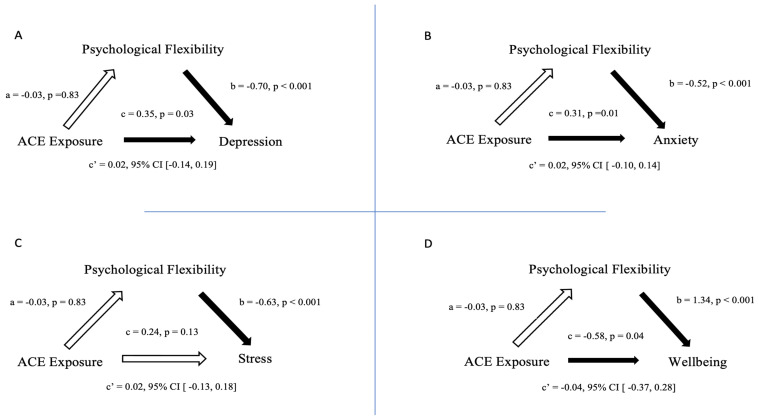
Mediation Analyses. Each quadrant represents a mediation analysis with ACE Exposure (ACE Questionnaire total) as a constant predictor variable with Psychological Flexibility (PsyFlex outcome) as a constant mediator variable. The outcomes change as follows: (**A**) Depression (DASS-21); (**B**) Anxiety (DASS-21); (**C**) Stress (DASS-21); and (**D**) Wellbeing (WEMWBS). Note: “c” refers to bivariate effect of predictor on outcome; “c’” refers to bivariate effect of predictor on outcome while controlling for the mediator. Arrows: hollow (white) arrows indicate a non-significant mediation effect, with filled (black) arrows indicating a significant relationship.

Mediational analyses with psychological flexibility as a mediator of the relationship between ACE (predictor) and psychological distress (DASS-21 scales) at Time 2 (outcome) were carried out. Bivariate correlations are reported in Table 2. Results from the mediational analyses are illustrated in Figure 1. In all the mediation models ACE was not a significant predictor of Psychological Flexibility (Path a), b = −0.03, t (228) = −0.22, *p* = 0.83. Psychological Flexibility was a significant strong negative predictor of all three outcomes of psychological symptoms (path b)—depression, b = −0.69, t (228) = −11.78, *p* < 0.001; anxiety, b = −0.52, t (228) = −10.72, *p* < 0.001; stress, b = −0.63, t (228) = −10.63, *p* < 0.001. ACE was a significant positive predictor of outcomes for depression and anxiety (path c)—depression b = 0.35, t (228) = 2.21, *p* = 0.03; anxiety, b = 0.31, t (228) = 2.45, *p* = 0.01 but not for stress—b = 0.24, t (228) = 1.52, *p* = 0.13. There was no significant relationship between cumulative ACE scores, and the outcome on Psychological Flexibility (Path a). However, Psychological Flexibility was a significantly positive strong predictor of wellbeing (Path b), b = 1.34 t (228) = 13.34, *p* < 0.001, and ACE was a significant negative predictor of wellbeing (path c), b = −0.58, t (228) = −2.02, *p* = 0.04.

## 4. Discussion

The objective of this study was to investigate the longitudinal profile of psychological wellbeing and distress, during the COVID-19 pandemic, to investigate whether a history of self-reported ACE related to a person’s wellbeing during the COVID-19 pandemic. The anticipated decrease in mental health and wellbeing due to COVID-19 will likely peak in the mid and post-pandemic phases and persist for years to come [4,22,65]. This longitudinal study investigated mental health and wellbeing changes from the beginning of the pandemic (March 2020) and again approximately ten months into the pandemic (January 2021), with a specific interest in the response profile and self-reported stress of people who had experienced ACE, compared to those without. This study hypothesized that psychological distress would increase over time for those with ACE, relative to the control population (those with no ACE). In addition, it was hypothesized that those with No ACE would have significantly lower distress at Time 2 compared to those with ACE, based on the stress sensitization hypothesis. Finally, this study also aimed to investigate whether self-reported Psychological Flexibility was a protective mediating factor on psychological distress, wellbeing, and the ACE-Distress relationship. Recent findings related to COVID-19 and historical research show that the mental health fallout from COVID-19 will disproportionately impact those already vulnerable in society [65,66,67,68]. This study supports previous research showing that those who have experienced ACE report greater psychological distress over time during the current COVID-19 pandemic [25,26,32,36].

### 4.1. Longitudinal Change between and within Those with ACE and Those without ACE in Their Mental Health and Wellbeing

Significant increases in mean scores were found in all three categories of the DASS-21 subscales (depression, anxiety, and stress) for individuals with Low ACE scores, with those for depression and stress being of medium effect size (*d* = 0.5) and anxiety being of small effect size (*d* = 0.2). For those with High ACE, only Depression scores increased significantly, and were of medium effect size (*d* = 0.5). For those with No ACE, there were no significant increases over time, indicating that those with No ACE may have had better psychological adjustment over time, as scores remained more stable for the duration of this longitudinal study. These findings align with previous research into the SARS pandemic, where pre-outbreak traumatic experiences were a significant predictor of post-SARS outbreak levels of depression symptoms, even after three years [20]. When taken together, these findings support Robinson et al. [69], who reviewed 65 longitudinal studies and found increases in depression scores over time to be more pronounced than other measures of distress such as anxiety. Although increases in depression were not significant for those with No ACE, this increase is something to be cognizant of in future investigations.

There were no significant differences found between the groups at Time 1 on any of the measures of Distress. However, significant differences were found over time between groups in reported levels of depression and stress but not anxiety. Planned contrasts revealed support for our hypotheses, with increased depression scores over time for those with No ACE being significantly lower, relative to the increase reported in the Low and High ACE categories. In terms of Stress scores over time, there were significantly lower relative change for those with No ACE than those within the Low ACE category, but not the High ACE category. This would suggest that the number of ACE alone is not the sole factor responsible for this between group difference in psychological distress, and that not all facets of psychological distress change equally, as highlighted by Robinson et al. [69].

These findings have clinical significance. They demonstrate that both those with and without ACE initially responded similarly and most likely adaptively to the threat and uncertainty of living alongside COVID-19, with both reporting similar levels of distress. Notwithstanding, for those with ACEs, psychological distress continued to increase significantly over time with the highest rate of change reported for the Low ACE group, and highest elevations observed in the High ACE group. Clinically, this may be beneficial in terms of psychological formulation, and co-formulation, and the consideration of ACE as predisposing and perpetuating factors relative to prolonged stress exposure. Further clinical consideration could also be given to implementing or integrating interventions which include psychological flexibility, as this was shown to mediate psychological distress in the context of ACEs.

For everyone equally, those with and without ACE, self-reported wellbeing significantly decreased over time. This may be expected due to the social and lifestyle restrictions in place at that time due to public health measures, yet contextually, this study took place during equivalent governmental lockdowns, i.e., Level 5.

These findings support those of Shevlin et al. [70] who highlight that the differential findings in many studies are due to responses being treated as homogeneous rather than heterogeneous. Furthermore, it supports the use of looking at temporal changes over more extended periods than done in many previous studies, whereby changes were only looked at up until June 2020 [69]. Furthermore, these results support Hammen’s et al. [24,44] stress sensitization model, which posits that adversities in early childhood sensitize individuals to subsequent proximal stress and may increase the risk for psychopathology in the face of future stressful life events.

### 4.2. ACE-Distress Relationship, ACE-Wellbeing Relationship and Psychological Flexibility-Mediational Analyses

Mediational analyses revealed Psychological Flexibility as a strong significant negative predictor of all three DASS-21 outcomes, while ACE score was a significant positive predictor of anxiety and depression, but not stress. ACE total score did not significantly predict Psychological Flexibility. While Psychological Flexibility did reduce the effect ACE exposure had on prolonged distress outcomes, it was not significant and did not mediate this relationship. Concerning wellbeing, Psychological Flexibility was a significant positive predictor, and also indirectly reduced the negative effect of ACE exposure on wellbeing with a small mediation effect observed.

In terms of future research, for people with or without ACE, fostering Psychological Flexibility skills through evidence-based interventions could be used to improve wellbeing, reduce psychological distress, and increase resilience against prolonged stressors [14,19,71]. Research could prospectively investigate psychological interventions such as these, which may mitigate this ACE- Distress/Wellbeing relationship.

### 4.3. Limitations

This study has limitations that can be built upon for future research. For example, the convenience sample recruited for this study led to an unequal gender balance, with 88% of this study being female. Additionally, this study required participants to complete surveys electronically and to have access to the internet, which is a barrier for people with complex and enduring mental health difficulties [45]; thus, this study may be prone to sampling bias. Lastly, this study does not account for other psychosocial stressors or contextual factors which people with ACE may experience, and while increasing and incremental psychological distress was associated with ACE, future research should aim to investigate and understand psychosocial inequity alongside psychological outcomes.

### 4.4. Conclusions

This longitudinal study aimed to access the longer-term effects of COVID-19 on mental health and wellbeing, with a particular interest in those who have experienced ACE relative to those who have not. This study shows on a group level, before any stratification, that there is a statistically significant increase in Depression, Stress, and Anxiety, and reduction in psychological well-being over time. However, when stratified by those who report ACE and those who do not, the effect of increased psychological distress is not present for the non-ACE group, though significantly reduced psychological wellbeing remains. For those who report ACE, there is significantly higher levels of psychological distress over time as well as reduced psychological wellbeing, with increased ACE relating to increased psychological distress. This study further shows that Psychological Flexibility has a strong negative relationship to psychological distress and mediated the relationship between ACE and psychological wellbeing.

Before COVID-19, the mental health services in Ireland were underfunded, and only 6% of the overall health budget was dedicated to mental health, half of what other countries such as the United Kingdom and New Zealand contribute. With such limited resources, it is of utmost importance to support those most vulnerable to the enduring effects of COVID-19. Therefore, in line with the findings of this study, those with ACEs should be considered within this category and their needs responded to accordingly.

As supported by our findings, examples of this could be through evidence-based interventions in the community and clinic that utilize Psychological Flexibility as a core concept, such as ACT, Mindfulness, or positive psychology-based interventions. Service-level additional measures could be adopted from other countries, i.e., Scotland National Health Service, such as improved screening [72]; and introducing a national trauma training programme in all settings (schools, prisons, police force, hospitals, etc.). Emanuel and colleagues [73] note that it is now the ethical responsibility of those in government to uphold the principle of reciprocity, whereby society should now return the goodwill shown by individuals during the COVID-19 pandemic in following public health guidelines by providing adequate medical, social and psychological support as needed during and after the initial phases of the pandemic.

## Figures and Tables

**Table 1:** Mean and Standard Deviations across psychological wellbeing variables over time, stratified by ACE grouping.

	Total Cohort (*n* = 231)						No ACEs (*n* = 75)						Low ACE 1-3 (*n* = 110)						High ACE 4 or More (*n* = 46)					
	Time 1		Time 2		*p*	d	Time 1		Time 2		*p*	d	Time 1		Time 2		*p*	d	Time 1		Time 2		*p*	d
	M	SD	M	SD			M	SD	M	SD			M	SD	M	SD			M	SD	M	SD		
**Depression**	5.36	4.64	7.19	5.35	**0.001**	−0.409	5.24	4.24	5.93	4.77	0.139	−0.173	5.42	4.54	7.75	5.48	**0.001**	−0.533	5.41	5.52	7.87	5.66	**0.001**	−0.485
**Anxiety**	3.11	3.38	3.60	4.20	**0.046**	−0.132	3.12	3.42	2.85	3.55	0.442	−0.089	2.95	2.98	3.80	4.20	**0.032**	−0.208	3.50	4.18	4.33	5.02	0.112	−0.239
**Stress**	6.61	4.66	7.85	5.11	**0.001**	−0.265	6.69	4.20	6.69	5.05	1.00	0.000	6.59	4.77	8.61	5.24	**0.001**	−0.438	6.50	5.18	7.91	4.60	0.058	−0.287
**Wellbeing**	46.88	8.32	43.80	9.58	**0.001**	−0.367	47.89	7.17	45.55	9.36	**0.001**	0.266	46.65	8.03	43.35	9.80	**0.001**	−0.404	45.78	42.02	42.02	9.10	**0.001**	0.452

Note: M = Mean; SD = standard deviation; *p* = significance value; d = Cohen’s d; bolded = significant < 0.05.

**Table 2:** Bivariate correlations among variables for mediation analysis.

Variable	1	2	3	4	5	6
1 ACE Questionnaire	1	-	-	-	-	-
2 PsyFlex	−0.03	1	-	-	-	-
3 DASS-21: Depression	0.35 *	−0.70 ***	1	-	-	-
4 DASS-21: Anxiety	0.31 **	−0.52 ***	0.65 ***	1	-	-
5 DASS-21: Stress	0.24	−0.63 ***	0.78 ***	0.72 ***	1	-
6 Well-being	−0.58 *	1.34 ***	−0.77 ***	−0.58 ***	−0.70 ***	1

Note: * *p* < 0.05; ** *p* < 0.01; *** *p* < 0.001.

## Data Availability

The data presented in this study are available on request from the corresponding author.
